# It Won’t Be Easy: How to Make Universal Pharmacare Work in Canada

**DOI:** 10.15171/ijhpm.2019.82

**Published:** 2019-10-08

**Authors:** Steven Lewis

**Affiliations:** ^1^Simon Fraser University, Burnaby, BC, Canada.; ^2^University of Saskatchewan, Saskatoon, SK, Canada.

**Keywords:** Universal Coverage, Pharmaceuticals, Cost Containment, Implementation

## Abstract

One of the glaring gaps in Canada’s universal healthcare system is the low level of public financing of prescription drugs - 42.7% of total spending in 2018. At the federal level there is renewed interest in moving towards universal coverage, supported by a recently commissioned report on how to achieve it. It will take superb political navigation to extract Canadian pharmaceutical policy and practice from the grasp of interests that profit handsomely from the status quo. This perspective suggests the conditions under which a genuinely fair, effective, and efficient pharmacare plan can emerge.

In World War I over 66 000 Canadians died in the killing fields of Europe. In 1980 the Canadian embassy in Iran executed a daring strategy to smuggle eight at-risk diplomats out of the country. In 2005 Canada became the fourth country, and the first outside of Europe, to legalize same-sex marriage. In 2018 Canada resettled more refugees than any country in the world.^1^ The 2019 US News and World Report country rankings place Canada 3rd overall and #1 for quality of life.^2^ It is an admired nation,^3^ generous and tolerant, an oasis of openness and multiculturalism thus far largely resistant to the toxic nativisms on the rise elsewhere. Canada has done hard things few other countries have managed to do. Yet it has not done what many other countries have done easily. Exhibit One is universal pharmacare.

Pharmacare is medicare’s Achilles heel and unfinished business, Canada’s *Sagrada Familia* cathedral, its cornerstone laid in Barcelona in 1882 and still undone. In 2018 only 42.7% of prescription drug expenditures were publicly financed.^4^ The political reasons for the absence of progress towards pharmacare are varied and complex, and for reasons of space cannot be recounted here.^5,6^ Suffice it to say that no government, provincial or federal, has won office by promising it and none has been defeated because it has not. The rationale in favour is rock solid, as it has always been.^7^ At least 3 million Canadians do not get essential medicines. (They are politically powerless, too busy deciding between groceries and drugs to mobilize.) New Zealand, with fewer people than British Columbia, pays far less for both patented and generic drugs than Canada.^8^ For patented medicines Canada has historically outsourced its bargaining, accepting the median price among seven comparator countries (to be expanded to 11 in 2020). Per capita spending on drugs is 43% higher than the Organisation for Economic Co-operation and Development (OECD) average.^9^ Yet for all these stains on the national honour, he absence of universal pharmacare has for the most part occasioned no more than a periodic we-really-ought-to-get-to-that conversation while the port is poured and the cigars lit.

The injustice is troubling, but the hypocrisy is worse. The governments that have rejected universal public coverage provide it for their own employees in the form of extended health benefit plans. Millions of vulnerable citizens – the non-unionized working poor, the not quite indigent elderly, struggling small business owners – pay taxes to help pay for the drugs of people who work for the governments that will not pay for theirs. Canadians are good at irony.

Vacuums create forces to fill them. In the absence of a universal plan to cover an obviously essential service, employers got into the healthcare business, their participation greased by incentives difficult to refuse. Employer costs are tax deductible and hence publicly subsidized. The rest can be passed onto consumers in the form of higher prices. Employees get subsidized access to drugs, and the employer-sponsored benefits are tax-free to individuals. Private insurers are like the casinos that employ the dealers and accountants, take the house cut, and make shareholders happy. There is the illusion of benefits management but no one – not the employee with a use it or lose it entitlement; not the employer anxious to retain good employees and maintain calm labour relations; not the insurer for whom raising premiums is a better option than to risk losing clients by playing bad cop – has any incentive to promote rigorously evidence-based utilization. The whole scheme is a textbook case of moral hazard.

Just maybe it is finally pharmacare’s time. Commissioned by the federal government, *A Prescription for Canada: Achieving Pharmacare for All*^10^ (hereinafter the Hoskins Report) makes 60 recommendations on how to achieve universal drug coverage. One can quibble here and there, but the report is clear and principled, and acting on all or most of what it prescribes would make things better, not least because it would be difficult to make them worse. But it is less a blueprint than an artist’s rendering, and as a film it edited out some graphic scenes and stern language to achieve a G rating. It ably describes the train wreck that is the Canadian drug sector but is silent on the forensics of why it went off the rails. The status quo did not emerge by spontaneous generation. It is the product of influence and design, horse trading and political calculation, the sacrifice of good government on the altar of peace and order. The interests that created and profit by it are still in play, entrenched and cunning, and their legions are massed on the banks of the Rubicon to defend it.

Yet for all the rot of current arrangements and the impeccable logic of admitting pharmacare to the medicare tent, it is not a Nadal-in-the-French Open sure win. Canada’s drug sector is a mess because private and particular interests supersede the public interest. Current policy and practice make a mockery of the principles of medicare (medical necessity, accessibility, and comprehensiveness are glaring casualties), favour the strong over the weak, the prosperous over the poor, the suppliers over the patients. A lot of health and a great deal of money are at stake.

The Hoskins Report vision for pharmacare will not see the light of day because it rights wrongs, helps the disadvantaged, improves prescribing, or lowers prices - though it would do all of the above. It will emerge in recognizable form only through brave and artful politics at all levels, and carefully designed processes that will translate good intentions into good practice. What starts out with hope and promise and inspiring language can end up in self-parody. The Chinese Constitution declares that all nationalities are equal and commits the state to the protection and preservation of human rights.

In the Hoskins Report the words “values” and “fairness” appear 10 times each – invariably to salute Canadians’ world class commitment to justice – and the word “interests” only once, in passing. Like the rest of medicare, pharmacare is redistributive, and if sensibly designed it will be anathema to those who profit from the prevailing illogic and inefficiency. They will energetically protect their interests, either by stalling pharmacare in its tracks or chipping away at the implementation plan until it no longer resembles the original. They are sophisticated, well-connected, and rich. They know how to push politicians’ buttons and convince the public that no government plan could treat them as well as the pharma-private insurance alliance. To succeed, the architects of universal pharmacare must produce a clear, brave, and sustained counter-narrative. Getting it right is a job for vertebrates.

Governments must of course design pharmacare well, but successful implementation is in the hands of five major political constituencies. First among them are physicians, the gatekeepers to prescription drug use and the *de facto* stewards of the approved inventory. Visions of improved quality, prudent use, and better value for money will shatter into shards of disappointment unless doctors individually and collectively work to eliminate inappropriate prescribing, accept generics and cost-effective therapeutic equivalents as the defaults, and promote responsible and prudent use to their patients. They must get their pharmaceutical education from independent peer reviewed science and unconflicted mentors rather than beguiling drug company promotions. They must partner with regulators, professional associations, specialty societies, educators, and governments to ensure that perfection does not become the enemy of the good, and resist the temptation to declare pharmacare a disaster when difficult formulary decisions are made, or when efforts are made to curb overuse.

Getting physicians on board will be a challenge, but arguably less difficult than navigating the economics and politics of retail pharmacy, a business nested within and owned by much larger businesses dominated by chains. The pharmacy is not the principal enterprise, and it occupies a tiny fraction of the floor space in large commercial enterprises that now include grocery stores and Walmarts. The interests and culture of the larger enterprise invariably infiltrate the pharmacy.

To work as the Hoskins Report intends, universal pharmacare requires a non-commercial, non-profit-maximizing ethos that views drugs as public goods rather than free market commodities. It relies on pharmacists who are experts on drugs but agnostic about their use. Drugs are already the most commodified healthcare sector. By paying pharmacists for dispensing, governments have abetted a consumerist drug culture. The easy money comes from filling a bottle and attaching a label, not from questioning dubious prescriptions and educating doctors and patients. Any system that rewards volume and discourages meaningful professional engagement with patients and other healthcare providers is a threat to a well-functioning pharmacare. Changing professional culture is difficult under any circumstances, but impossible without eliminating the perverse incentives embedded in the retail drug economy.

The third constituency is private drug insurance, the 100 000 private plans pharmacare is designed to replace. The sector is an enormously over-populated and inefficient house of cards. If it disappears, people will lose their jobs, like auto workers replaced by robots and bank tellers by ATM machines. Obsolescence is the cruel side effect of progress. If we kept every once-useful process or technology in service, offices would be alive with the clackety-clack of IBM Selectric typewriters.

The Hoskins Report is here it is too deferential, polite to the point of self-contradiction. “National pharmacare should offer comprehensive and affordable coverage so that no one will need supplementary private drug insurance.” Quite so, but then: “Nonetheless, individuals should be able to purchase private insurance to cover any out of pocket costs associated with pharmacare (such as copayments) and for drugs not listed on the national formulary.”

It is disingenuous to offer an irrational choice. There is no rational market for insurance to cover the recommended copayments of $5 per prescription and $100 annually. So the role of private insurance must be to cover drugs not listed on the national formulary. If the formulary is well-constructed by disinterested experts, these excluded drugs have failed to meet reasonable standards of efficacy and/or cost-effectiveness. If the standards are too high, reasonable people will deem coverage inadequate and pharmacare will die by a thousand re-emerging private plans. If the standards are sound and evidence-based, and if there is the safety valve of a separate pool to cover some experimental, high cost, and rare condition drugs, what would one buy insurance for? By definition, access to drugs unlikely to be effective and/or extraordinarily expensive.

Some excluded drugs will have a (usually tiny) chance of working where all others have failed, a final tilt at a daunting windmill. It is both foreseeable and understandable that some people in some circumstances will want these drugs listed on either the regular or special formulary. The contested decisions will revolve around drugs that might be helpful, but not helpful enough to pay for even from the special cases fund.

No public (or private) plan can fund every drug in every circumstance at any cost. An excellent public school system does not provide one-on-one tutoring for every special needs student. No government builds divided highways to every community even though doing so would prevent some harms. A well-meaning physician might propose and a patient might be willing to try an excluded drug once all other option have been exhausted. But it would be sheer folly to design universal pharmacare around individuals’ decision criteria in dire circumstances.

Regardless, it makes little sense either to offer or buy insurance for these anomalous cases. The insurer would have to demand high premiums, exclude pre-existing conditions, and develop elaborate case-by-case adjudication criteria to minimize risk. The buyer would need a lot of disposable income, be willing to pay the high costs of insuring against very low probability events, and agree to the insurer’s terms and conditions. It is difficult to imagine a scenario where either the insurer or the buyer is not seriously miscalculating expected benefits. Patients would almost always be better off self-insuring: simply pay for the drugs out-of-pocket. For very expensive drugs, neither option is available to the non-rich and those disinclined to chase miracles at great cost, but in the grand scheme of things this minor inequity is hardly fatal to the fairness of a soundly built public plan.

The report ultimately shrinks from its own diagnosis, reluctant to cut down the dying tree looming ominously over the roof. Instead it offers private insurance a bone: “They [the employers] will have the financial room to offer other health benefits to their workers (for example, mental health and wellness services, physiotherapy, dental and vision care)…” Here is where tactful silence would have been preferable. The rationale for universal pharmacare applies equally to other services not covered by medicare, such mental health, physiotherapy, dental, and vision care. All are medically necessary, many go without to their detriment, and in the case of dental^11^ and vision care,^12^ costs are rising quickly and supplier-induced demand (upselling) is common. To enhance population health and the comprehensiveness of primary care, improving access to mental health services is at least as important as universal pharmacare. It makes no sense to endorse private, employer-sponsored insurance for the gander after explaining why it is inappropriate for the goose.

Private drug insurance, like coal mines and tobacco companies, is the problem to be solved, not a precious legacy to be repurposed. There is no point putting it on expensive life support once it can no longer survive on its own. Rather than flinch and prop up a doomed industry, public policy should bid it farewell and support the transition of displaced employees to more useful roles.

The fourth, and most sophisticated and powerful group is pharma. Canada now pays among the highest prices in the world, and with a fully empowered single negotiator – the already-existing Pan-Canadian Pharmaceutical Alliance of governments recommended by the Hoskins Report - there is a reasonable prospect of saving several billion dollars a year.^13^ (Currently its recommendations are non-binding on provinces and territories.) There is a precedent: provincial governments slashed unconscionably high generic drug prices dramatically a few years ago. The more difficult battle will be to keep marginal drugs out of the formulary. Canada has excellent drug assessment expertise, now nationally coordinated through the Canadian Agency for Drugs and Technology in Health’s common drug review protocol. Pharma is adept at enlisting prominent physicians and patient advocacy groups in the permanent campaign to secure coverage of expensive drugs with modest health benefits and expand the pool of patients for whom use is approved. The pharma war chest is effectively unlimited; companies caught red-handed in deceitful marketing practices and price-fixing appear to shrug off billion-dollar plus fines with equanimity as just another cost of doing business.^14^

Ideally drug companies would adjust their practices to help make pharmacare work, but it is a big ask. Their current business model owes much of its success to me-too drugs, mischievous litigation to extend the life of patents, massive marketing campaigns, excess utilization, and price maximization. It is unrealistic to anticipate an industry-wide epiphany followed by reinvention. Pharma will cleave to its model until it has been shown that the politics can withstand the inevitable torrent of misinformation, special pleading, and orchestrated outrage against rational decisions and tough bargaining.

Governments should neither deliberately alienate pharma nor secure its affections through bad policy. Little New Zealand is indifferent to pharma’s wishes and is willing to live without a significant R&D presence. If drug-makers threaten to shut down their Canadian R&D, currently about a billion dollars annually,^15^ there will be a loss of jobs and perhaps a foregone discovery, although I cannot find evidence that Canadian research sponsored by multinationals has been primarily responsible for a single breakthrough drug in the past 50 years. The R&D jobs are clustered in metro Montreal and Toronto, obviously important politically. But the loss of pharma R&D dollars need not mean a net loss in total R&D spending.

In a tax-funded pharmacare, costs will shift from businesses to government. Universal pharmacare should be cost-neutral for business, with the money previously spent on private plans harvested as taxes and invested in R&D, evaluation, and education based on a public interest model. This would be a boon to researchers and a catalyst for quality improvement. Successful pharmacare is a scientific, cultural, and behavioural transformation that cannot be permanently sustained without adequate investment on all three fronts.

The fifth and most critical constituency is the public. Two-thirds of Canadians, largely middle class, are covered by private plans, which among other things accounts for the muted political pressure to implement a universal plan. While the public supports universal coverage, there are concerns about a reduction in benefits and choices.^16^ It is conceivable – probably inevitable – that a national formularly will exclude some drugs now covered in some private plans. Citizens cannot be fair-weather pharmacare supporters, demanding prudent stewardship of resources and consumption in all cases but their own, or both generous coverage and lower taxes. They must be open to the notion that existing plans are inefficient and that there are good reasons for a universal formulary to exclude drugs to which they currently have access. They must stand behind governments that stand up to extortionist prices. Allegiance to pharmacare’s principles and coverage criteria cannot dissolve at the first appearance of heart-tugging individual cases.

Canada need not settle for an ascetic, minimalist pharmacare. It can afford a generous program. The need for healthcare in general, and costly drugs in particular, is unevenly distributed. Those with conditions for which there is no cheap and effective treatment should not be left out in the cold by pharmacare. Healthcare is a right and a compassionate enterprise that cannot adhere to strict utilitarianism. It will cost tens of thousands of dollars a year to treat some and $100 a year to treat others, based on need and the state of science. Special circumstances warrant special consideration. But the greatest good for the greatest number remains the ethical foundation of public programs even if it ought not to be applied too strictly. Sometimes when the heart says yes the head must say no, not for lack of empathy, but because more good can be had elsewhere.

There may well have to be compromises in formulary design to acquire and maintain the support of large segments of the population attached to their current plans. There will be fewer hard choices to make if doctors and pharmacists mobilize to promote evidence-based practice and pharmacare harvests the dividends of lower prices and prudent use. Billions are now wasted on overuse: opioids, benzodiazepines, older people on ten or more medications. Some overuse is not just low value, but harmful, incurring additional health spending.^17,18^ Perhaps a tenth of elderly admissions to hospital result from adverse drug reactions.^19^ The more prudent overall drug use, the more money available for new, plausibly effective, but very expensive drugs for more people, at the very least on an experimental basis to ascertain their true value over time.

A high-functioning pharmacare plan is what microbiologists call a fastidious organism: it needs a specific culture to thrive. If the culture is inhospitable, the organism will be disfigured. The Affordable Care Act in the United States has extended health insurance to some 20 million people, but to get it passed the Obama administration made enormous concessions to pharma and the private insurance industry, leaving massive inefficiencies in place.^20^ Many of Canadian medicare’s shortcomings are traceable to the founding compact with physicians that enshrined both independent contractor status, and fee-for-service as the dominant payment method. It can take a very long time to recover from deals that looked good at the time.

Universal pharmacare is a formidable design challenge that demands the balance of a gymnast, the patience of a kindergarten teacher, the calculating discipline of a poker player, and the precise teamwork of a pit crew. Policy-makers, doctors, and pharmacists must do their parts to make good on its promise. It would be a bonus if pharma changed its ways. But much depends on sensible and smart citizens and patients committed to prudent use, supportive of sound coverage rules fairly applied, accepting of tough calls in tough cases, and resistant to seductive marketing and the promise of a quick fix. This is as much a civic as a technical challenge, dependent on the politics of good policy-making, the balancing of interests, and above all a shared commitment to the public good. Ask not (only) what pharmacare can do for you; ask what you must do for pharmacare to make it a unifying, just, and health-enhancing program.

## Ethical issues

Not applicable.

## Competing interests

Author declares that he has no competing interests.

## Author’s contribution

SL is the single author of the paper.
